# Necrotising enterocolitis and mortality in preterm infants after introduction of probiotics: a quasi-experimental study

**DOI:** 10.1038/srep31643

**Published:** 2016-08-22

**Authors:** Noor Samuels, Rob van de Graaf, Jasper V. Been, Rogier C. J. de Jonge, Lidwien M. Hanff, René M. H. Wijnen, René F. Kornelisse, Irwin K. M. Reiss, Marijn J. Vermeulen

**Affiliations:** 1 Erasmus MC, Department of Paediatrics, division of Neonatology, Rotterdam, 3000 CB, The Netherlands; 2 Erasmus MC, Department of Hospital Pharmacy, Rotterdam, 3000 CB, The Netherlands; 3 Erasmus MC l, Department of Paediatric Surgery, Rotterdam, 3000 CB, The Netherlands

## Abstract

Evidence on the clinical effectiveness of probiotics in the prevention of necrotising enterocolitis (NEC) in preterm infants is conflicting and cohort studies lacked adjustment for time trend and feeding type. This study investigated the association between the introduction of routine probiotics (Lactobacillus *acidophilus* and Bifidobacterium *bifidum;* Infloran^®^) on the primary outcome ‘NEC or death’. Preterm infants (gestational age <32 weeks or birth weight <1500 gram) admitted before (Jan 2008–Sep 2012; n = 1288) and after (Oct 2012–Dec 2014; n = 673) introduction of probiotics were compared. Interrupted time series logistic regression models were adjusted for confounders, effect modification by feeding type, seasonality and underlying temporal trends. Unadjusted and adjusted analyses showed no difference in ‘NEC or death’ between the two periods. The overall incidence of NEC declined from 7.8% to 5.1% (OR 0.63, 95% CI 0.42–0.93, p = 0.02), which was not statistically significant in the adjusted models. Introduction of probiotics was associated with a reduced adjusted odds for ‘NEC or sepsis or death’ in exclusively breastmilk-fed infants (OR 0.43, 95% CI 0.21–0.93, p = 0.03) only. We conclude that introduction of probiotics was not associated with a reduction in ‘NEC or death’ and that type of feeding seems to modify the effects of probiotics.

Necrotising enterocolitis (NEC) is the leading cause of death among very low birth weight (VLBW) infants^1^,^2^,^3^. Up to half of all infants who develop NEC require surgical treatment^4^,^5^. Survivors of NEC are at high risk of serious short and long-term complications, including neurodevelopmental impairment^6^,^7^. NEC is a multifactorial disease with a crucial role for the microbiome and immaturity of the gastrointestinal tract and immune system^8^. Probiotics, also naturally present in breast milk, are increasingly being used in an attempt to prevent NEC^9^.

Meta-analyses of over 20 randomised controlled trials (RCTs) including over 5000 patients indicate that supplementation of probiotics safely reduces NEC incidence and all-cause mortality in very preterm infants^10^,^11^. However, many questions and issues still remain^12^. The meta-analyses were summaries of different probiotic products and treatment protocols and did not allow drawing conclusions on the most beneficial strains and strategies. The recent large RCT on Bifidobacterium (B.) *breve* illustrated that probably not every probiotic strain has an effect^13^. Another issue is that probiotics are not licenced as drugs in most countries^14^. As drug license is also lacking in the Netherlands, we requested special permission from the Dutch Health Care Inspectorate (DHCI, equivalent to the US FDA) for implementation of probiotics as standard care for the prevention of NEC in our neonatal intensive care unit (NICU). Like several other centres around the world, we chose Infloran^®^ (with the combination of Lactobacillus (L.) *acidophilus* and B. *bifidum* strains) but realised that the beneficial effect of this specific product was only shown in one registered RCT^15^.

To date, evaluation of clinical use of L. *acidophilus* and B. *bifidum* has been communicated at scientific meetings only^16^,^17^,^18^. These studies concerned small populations and used analyses without adjustment for relevant confounders such as type of feeding and underlying time trend. We therefore designed a large observational study and hypothesized that the incidence of NEC or death would decline over and above its underlying temporal trend after the introduction of routine probiotic supplementation in our NICU.

## Methods

### Study design and population

A “quasi-experimental” study was performed in the level IV NICU of the Erasmus MC, Rotterdam, The Netherlands. The study population consisted of two groups, defined by the moment of introduction of probiotics; one group represented those born before introduction of probiotics (Group 1; January 1, 2008 until October 1, 2012); the other those born after introduction of probiotics (Group 2; October 1, 2012 until January 1, 2015). Inclusion criteria were gestational age (GA) <32 weeks and birth weight (BW) <1500 grams. Outborn infants admitted to the NICU after the first day of life were excluded. Infants were transferred to level II centres as soon as they were clinically stable with continuous positive airway pressure (CPAP) or nasal flow, weighted >1000 gram and had reached a postmenstrual age of >30 weeks. On admission, all parents were informed on the use of anonymised demographic and medical data from the medical records for national registries and for evaluation of clinical practice. Because this study was a retrospective observational study using anonymized data collected during routine clinical practice, informed consent was not mandatory according to the Dutch Medical Research Involving Human Subjects Act (WMO). The Institutional Ethics Review Board of the Erasmus MC reviewed the study protocol and provided an exemption from formal ethical assessment (MEC-2013-409) based on the non-interventional design. The study was carried out in accordance with the current ethical guidelines for epidemiological research.

### Intervention

Infloran^®^ (SIT Laboratorio Farmaceutico, Mede, Italy) 250 mg capsules containing 10^9^ colony forming units (CFU) L*. acidophilus* (ATCC 4356) and 10^9^ CFU B. *bifidum* (ATCC 15696) were purchased^19^. Probiotic supplementation was started at the first enteral feed of at least 1 ml per bolus and was continued until 35 weeks postmenstrual age or until NICU discharge, whichever came first. A daily dose of one capsule was dissolved in 2 ml of (breast or formula) milk and given per nasogastric tube. If feeding amounts were still between 1 and 2 ml per bolus, an equivalent portion of the capsule was given. Infants with severe congenital abnormalities needing surgery did not receive probiotics prior to surgery. Probiotic supplementation was interrupted during periods of nil by mouth.

### Safety

Potential side effects (focussing on intolerance and probiotic sepsis) were recorded and reported to the DHCI. All causes of in-NICU deaths were individually reviewed, based on the medical record, including available post mortem pathology and microbiology reports. If none of these sources suggested probiotics as a cause of death we considered mortality unlikely due to probiotics. Routine colonisation and resistance surveillance included regular rectum and sputum bacterial cultures.

### Nutrition

Our enteral and parenteral nutrition protocol has been described previously^20^. If expressed breast milk was insufficiently available, preterm formula was supplemented (Nenatal Start^®^, Nutricia, Zoetermeer, The Netherlands). Donor breast milk was not available for routine use. A gradual change in policy was made after 2013 to limit exposure to formula feeding. Starting minimal enteral feeding within 6 hours after birth (thus giving formula feeding if breast milk was not yet available) was changed to waiting for colostrum until 12–24 hours after birth. From May until July 2014, 28 infants participated in the early nutrition study (ENS), a RCT comparing formula feeding to donor milk in addition to own mother’s milk^21^. All ENS participants were treated according the standard probiotic protocol and were included in the primary analysis. Twenty-four infants received the blinded nutritional intervention.

### Outcome definitions

*A priori,* the primary outcome was stipulated as the composite outcome of ‘NEC or death’ until day 120 of life. If the patient was transferred before this day, outcome was based on correspondence and personal communication of the other hospitals and on the neonatal follow-up data collected at the outpatient department. Four secondary outcomes were defined: NEC, mortality, and the composites of ‘surgical NEC or death’ and ‘NEC or sepsis or death’. NEC was defined as any episode meeting Bell stage 2 or 3^22^,^23^. Surgical NEC was defined as NEC requiring surgical intervention (also if the patient was too unstable to undergo surgery). The diagnosis of NEC was ascertained by having all individual patient files reviewed by two authors (NS and MJV) unaware of study group. We used the following additional definitions. Cases of spontaneous focal intestinal perforation, defined as isolated perforation in a normal-appearing bowel without features of NEC such as pneumatosis intestinalis or necrosis, were not classified as NEC^24^. Cases without pneumatosis but with (sub)total intestinal necrosis confirmed by surgeon (during laparotomy) or pathologist (tissue biopsy or post-mortem) were defined as NEC stage 3. Sepsis was defined as blood culture-proven late-onset (>72 hours after birth) sepsis with an elevated C-reactive protein concentration (>10 mg/L) occurring during NICU admission^25^.

### Data collection

Data were collected from the individual Electronic Health Record and the intensive care Patient Data Management System. Potential confounding variables included prenatal steroids (any dose/none); GA (preferably based on early obstetric ultrasound, otherwise on last menstrual period, in weeks); gender; mode of delivery (vaginal/caesarean section); BW Z-score^26^; and milk type (no milk/breast milk only/formula only/mix of formula and breast milk; ENS study milk). The category ‘no milk’ was applicable to infants who died or were discharged before the first enteral feeding. For ENS participants milk type was ‘study milk’ if they did not fulfil the criteria for ‘mixed’ and received the blinded intervention. Modification of the probiotic effect by type of milk feeding (different effect with breastfeeding versus formula feeding) has been suggested in earlier reports^15^,^27^. We therefore included an interaction term between the introduction of probiotics and milk type to assess whether the associations differed according to feeding strategy.

### Data analysis

We analysed patients according to whether they were born before (group 1) or after (group 2) the introduction of probiotics in our department, and not according to whether they actually received probiotics. We used Pearson’s chi-square test for parametric data and the Mann-Whitney U test for non-parametric data for comparison of baseline data. Unadjusted associations between the period before versus the period after probiotic introduction and each outcome were explored using univariable logistic regression analysis.

A multivariable model was used to investigate whether introduction of probiotics was independently associated with changes in the odds of developing the outcomes over and above the underlying temporal trends. Logistic regression models were developed with addition of variables allowing adjustment for temporal variation in the odds of developing the outcomes over time^28^. A seasonal pattern was reported by large cohort studies and analysis of our data confirmed a seasonal pattern in the odds of developing the primary outcome^29^,^30^. To include seasonality, quartile of birth was added as a covariate and was captured by dummy variables for quarters as appropriate. We also expected gradual changes in outcome over the 7 year study period. Besides measurable changes, such as breastfeeding and caesarean section rate, many other changes in obstetric and neonatal care may bias the association between introduction of probiotics and the outcome. Gradual changes over time were captured by addition of a continuous time variable, or B-splines in case of non-linearity of the underlying trend. This approach ensures that any differences in the odds of developing each outcome following the intervention, as identified by the models, were not in fact attributable to existing temporal trends or seasonal variation.

The models were built taking the following steps. The response to introduction of probiotics was assumed to behave as a sudden (‘step’) change, which was modelled using a dummy variable. The model was then built by adding the potential confounders. Next, we selected the optimal approach to account for possible underlying trends in the odds of developing each outcome over time: no time trend; linear time trend (modelled using a continuous time variable); or linear, quadratic, or cubic B-splines. Seasonality was assessed according to birth dates at the quarter-of-a-year level, modelled either via a categorical variable for quarter (i.e. Q1 to Q4) or a dummy variable for individual quarters, as appropriate. Adjustment for gender and gestational age was applied in all models regardless of statistical significance. The other confounders were dropped from the model one-by-one in a backward selection procedure, if statistical significance (using a cut-off of p < 0.1) was lacking^31^. We explored important differences between actual and model-predicted rates over time, and added in dummy variables for individual outliers, as appropriate. Selection of the optimal model at each step was informed by Aikaike’s and Schwarze’s Bayesian Information Criterion (AIC and BIC, respectively).

Analyses were undertaken using Stata SE version 13.0 (Statcorp, TX, USA). Medians, interquartile ranges (IQR) and odds ratios (OR) with 95% confidence intervals (CI) were reported. Statistical significance was defined at p < 0.05 (2-sided).

### Sample size

The cohort studied was a convenience sample, determined by the time period in which digital data were accessible. For multivariable interrupted time series regression analysis, samples that include 100 events and 24 time intervals are regarded large enough to assure adequate power^32^. With a total of 296 primary events in 28 quartiles, we considered the sample size amply sufficient.

## Results

### Baseline characteristics

**Figure 1:**
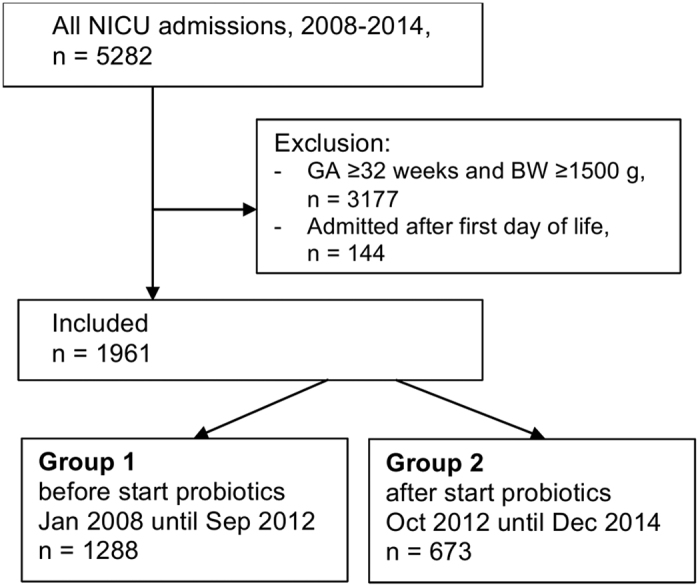
Study inclusion flow chart. BW, birth weight; GA, gestational age at birth; NICU, neonatal intensive care unit.

Figure 1 illustrates the selection and inclusion of 1961 infants, divided into group 1 (before introduction of probiotics, n = 1288) and group 2 (after introduction, n = 673). Baseline characteristics (Table 1) and clinical outcomes (Table 2) were tabulated for these groups. After routine introduction, 47 infants (7%) did not receive probiotics. This was because of discharge before probiotics were to be started (n = 15), death within the first few days of life (n = 12), or congenital malformations (n = 6). In 14 cases probiotics should have been started, but were omitted due to low assumed risk by the attending physician (GA < 32 weeks, but BW > 1500 gram; n = 13) or for no clear reason (n = 1).

**Figure 2:**
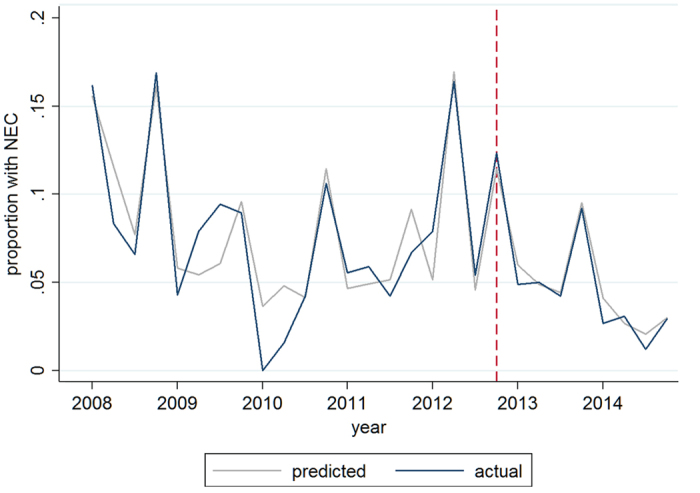
Quarterly incidence of NEC 2008–2014. The actual incidence, plotted in blue, reflects the observed proportion of infants developing NEC in the studied population, showing seasonality in NEC incidence. Red dotted line indicates moment of introduction of routine probiotics. The predicted incidence, plotted in grey, represents the estimated incidence of NEC by the interrupted time series model, with adjustment for gender, gestational age, birth weight Z-score, mode of delivery, prenatal steroids, (interaction between probiotic introduction and feeding type), type of feeding, seasonality and non-linear time trend.

A large difference in baseline characteristics is found in the type of feeding. In the period after introduction of probiotics, exclusive breastfeeding was more common (22% versus 4.9%), mainly at the expense of mixed feeding (75% versus 57%). Seasonality was observed in the incidence of NEC, which was generally highest in infants born in the fourth quartile of the year (Fig. 2).

### Unadjusted analysis

Table 3 shows that the primary outcome ‘NEC or death’ occurred in 15.5% of infants before and in 14.3% after introduction of probiotics, which was not significantly different (OR 0.91, 95% CI 0.69–1.18, p = 0.46). The incidence of NEC was significantly lower in the period after introduction of probiotics, with a decline from 7.8% to 5.1% (OR 0.63 (0.42–0.93), p = 0.02). No significant associations were observed in the other secondary outcomes.

### Adjusted analysis

**Figure 3:**
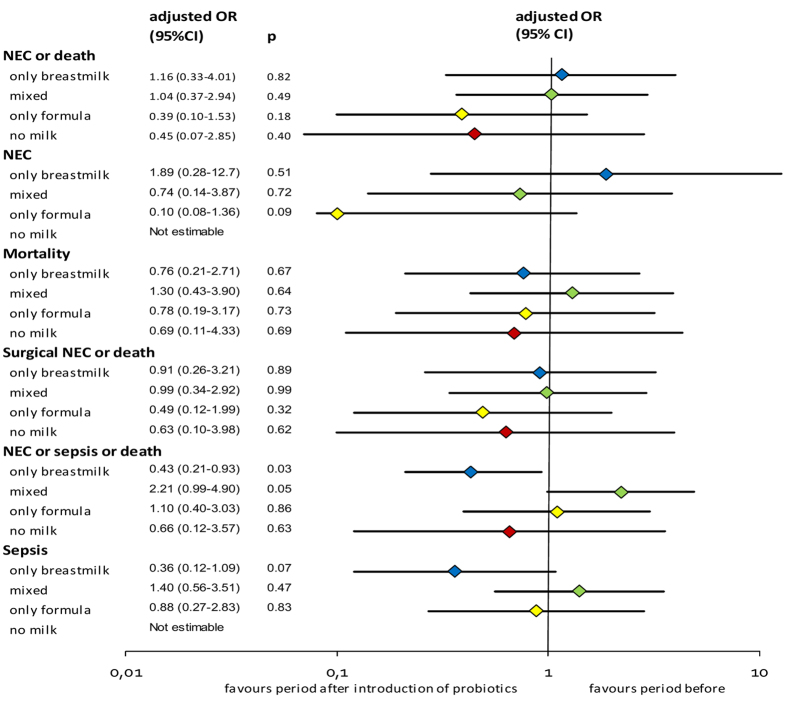
Adjusted associations between introduction of probiotics and each outcome according to feeding type. Shown are adjusted odds ratios (ORs) of developing the outcomes in the period after versus before introduction of probiotics for each feeding group. For each outcome a multivariable model was run, adjusted for gender, gestational age, birth weight Z-score, mode of delivery, prenatal steroids, the interaction between probiotic introduction and feeding and non-linear time trend. For more detailed output of the models: see Supplementary Table 1. Abbreviations: CI, confidence interval; NEC: necrotising enterocolitis. Definitions: NEC, necrotising enterocolitis ≥ stage 2; Surgical NEC, NEC requiring surgical treatment; Sepsis, defined as blood culture proven late-onset sepsis during NICU admission.

The interrupted time series model with adjustment for the confounders, seasonality and time trend showed that introduction of probiotics was not associated with a significant change in the odds of developing the primary outcome ‘NEC or death’ (Fig. 3 and Supplementary Table 1). Modification of the probiotic effect by type of milk feeding is suggested by the differences in odds between different feeding types. Having not been fed any milk during admission (‘no milk’) occurred rarely (2%) but it was very strongly associated with the outcome measures. As we suspected reversed causality (those who died early were not being fed) and the subgroup was small (see Table 1) we performed a sensitivity analysis. Exclusion of patients who had not been given any milk during admission or received ENS study milk did not have an impact on the association between introduction of probiotics and our primary outcome (data not shown).

Only in exclusively breastmilk-fed infants the introduction of probiotics was associated with a significant change in ‘NEC or sepsis or death’ (adjusted OR 0.43 (0.21–0.93), p = 0.03). To explore the association with sepsis alone we performed a post-hoc analysis, that showed no significant association between introduction of probiotics and sepsis (Supplementary Table 2). A second post-hoc analysis was performed to explore the role of probiotic treatment duration. Adding duration as a continuous covariate to the model indicated that longer duration was associated with lower odds for NEC or death (OR 0.95 (0.94–0.97)). Although adjustment for duration improved the model, the associations between introduction of probiotics and ‘NEC or death’ remained non-statistically significant in each type of milk group (Supplementary Table 2).

### Safety

Probiotic use was well-tolerated and no serious adverse events or deaths were considered to be related to probiotics use. In none of the blood or cerebrospinal fluid cultures Lactobacillus *acidophilus* or Bifidobacterium *bifidum* were encountered. No changes in colonisation and resistance patterns were observed.

## Discussion

Introduction of probiotics in the standard care for preterm infants at our NICU was not associated with a change in the odds of developing the primary outcome ‘NEC or death’. The unadjusted analysis suggested an association between introduction of probiotics and reduction of NEC incidence, but this was not statistically significant in the models adjusted for feeding type and underlying time trend. Introduction of probiotics was associated with reduced odds for the composite outcome ‘NEC or sepsis or death’ in exclusively breastmilk-fed infants only.

Lack of an overall beneficial effect of the introduction of probiotics on ‘NEC or death’ may be explained in several ways. First, introduction of L. *acidophilus* and B. *bifidum* may indeed be less effective in our setting than expected based on the literature. Generally, RCTs tend to overestimate the effect in real-life clinical practice, which may be due to selection of specific target populations and several other forms of bias^33^. On the other hand RCTs could also underestimate the true effect of probiotics due to cross-contamination between treated patients and controls in parallel treatment arms^34^. In our study, we did not compare outcomes between infants with and without probiotics in the same period and therefore cross-contamination was not considered an important bias. If cross-contamination has occurred, it reflects real-life practice and may have overestimated the effect in our study.

Confusingly, the product Infloran^®^ exists in different compositions (i.e. Lactobacillus *acidophilus* in combination with either Bifidobacterium *infantis* or with Bifidobacterium *bifidum*), depending on the country of distribution. Similar to Australia, New-Zealand and several centres in Asia and Europe we are using the latter combination (personal communication by manufacturer). Efficacy of exactly this product was shown in one RCT in 444 preterm breastfed infants in Taiwan (GA < 34 weeks, BW < 1500 gram)^15^. They found a significant reduction in ‘NEC or death’ (1.8% versus 9.2%; p = 0.002) and in NEC (1.8% versus 6.5%; p = 0.02) without any side effects. It is unknown whether probiotics have different effects in different ethnic groups, who may differ in composition of their microbiome. Recently, a beneficial effect of the same product was not confirmed by another Asian RCT, which found no reduction in NEC^35^. Their sample size (n = 60) was likely insufficient given the low background NEC incidence (3.4%) and low mortality (0%).

Secondly, low efficacy may be attributed to treatment duration, dosage or product inactivation. Although optimal treatment duration still needs to be established, probiotic treatment in the present study was relatively short. We aimed to treat until 35 weeks, when the risk of NEC has become low. Due to early transfer to other hospitals, treatment was stopped at a median postmenstrual age of 31 weeks. We speculate that some cases of NEC after transfer could have been prevented if treatment was prolonged. On the other hand, in a large observational study in Germany, probiotic treatment (L. *acidophilus* and B. *infantis*) given for only 14 days was beneficial^36^.

Furthermore, the dosage may not be optimal as dose finding studies have never been performed. In the two earlier RCTs a dosage of 0.5 capsule/kg twice daily was prescribed, whereas we did not dose per kg, but gave 1 capsule once daily^15^,^35^. Relative underdosing, compared to the earlier studies, may therefore have occurred only in infants weighing over 1 kg. Another potential cause of low efficacy may be found in inactivation of the probiotics^37^. We strictly controlled the cold chain (2–8° Celsius) from the pharmacy to the ward fridge, and mixed the dose to hand warm milk shortly before administration. Still, viability may have been at stake. Besides the quality checks of the manufacturer, checking for viability of the product was performed at the start of the study and at the end, but regular testing would have provided more information. Whether inactivated, dead or even degraded probiotic strains have beneficial effects in this context needs further study^38^,^39^,^40^.

Another factor that may lead to relative underestimation in our study may be found in the number of untreated infants. Analogous to intention to treat analysis we did not exclude any cases based on no or brief treatment. This approach has the strong advantage of reflecting clinical practice and creating a generalizable population, but leads to underestimation of the true treatment effect. After introduction, 47 (7%) infants did not receive any probiotics. We decided not to perform a sensitivity analysis with exclusion of these patients for the following reason. Untreated infants were those who died early (‘the worst’) and those who were stable enough to get discharged before treatment could be started (‘the best’). Selecting infants with similar risk profiles in group 1 was impracticable. Excluding non-treated infants from group 2 without exclusions from group 1 would actually have led to selection bias. In our opinion a valid comparison was only possible by excluding those with no milk (in both groups) or with unknown milk type (ENS study), which in a sensitivity analysis did not change our conclusions.

### Type of feeding

Interestingly, our study showed different associations in different feeding types, suggesting modification of the probiotic effects by the type of milk feeding the infants received. Although effect modification, or interaction, is biologically plausible, it has rarely been studied before in this context^27^. Most studies lacked information on type of feeding or studied populations with the majority receiving the same type of feeding^10^,^36^. Therefore, also the meta-analyses of RCTs were not able to stratify by type of milk feeding^10^,^11^. In our population formula and mixed feeding is still relatively common, which allowed for analysis according to type of milk feeding.

Only in exclusively breastmilk-fed infants we found a statistical significant association between introduction of probiotics and reduced odds for the composite ‘NEC or sepsis or death’. A post-hoc analysis showed that this was mainly explained by the trend of lower odds for sepsis in this group. To the best of our knowledge, the only report in this field considering effect modification by feeding type is a recent Austrian historic cohort study^27^. They compared VLBW Infants (BW < 1500 gram and GA < 34 weeks) in the period before (n = 233) and after (n = 230) introduction of probiotics and performed subgroup analysis per feeding type. They used Infloran^®^ with another composition, containing L*. acidophilus and* B. *infantis*. A significant association between probiotics and lower NEC incidence was only found in infants who were fed any breast milk (5.5% versus 11.1%, p < 0.05). The authors explained their findings by the symbiotic effect of the administered probiotics and (‘prebiotic’) oligosaccharides in breast milk. They did not correct for trend nor for interaction between probiotics and feeding type and did not include mortality in their outcome.

Although not statistically significant, the small odds ratio for developing NEC in the infants who are exclusively formula-fed (adjusted OR 0.10 (0.08–1.36), p = 0.09) may reflect a clinically relevant finding. This association with NEC is not seen in the other feeding groups and is therefore in contrast with the findings of the Austrian study. Of course it may be a chance finding in the current study. But if not, the most likely explanation could be that the infants who are at highest a priori risk for NEC and lack natural probiotic supply (and other immunological benefits) through breast milk would benefit most of probiotic supplementation. The interaction between probiotics and feeding type needs further study before any clinical decisions are made based on these data.

### Strengths and limitations

To our knowledge, this is the first published report on the clinical introduction of L*. acidophilus* and B*. bifidum* in a NICU setting and the first in this field to apply a statistical model adjusted for underlying temporal incidence trends. The detailed clinical data on a relatively large cohort allowed for adjustment for most relevant clinical risk factors including feeding type and effect modification.

The most important limitation of the study is the observational design. Random allocation to the intervention could have accounted more appropriately for (residual) confounding. Performing a RCT, however, was not feasible due to legal and ethical issues as put forward by others^41^. We therefore performed interrupted time series regression analyses, that is regarded as the strongest “quasi-experimental” approach and particularly useful when a (cluster) RCT is infeasible^28^,^42^,^43^. This method allows for adjustment for changes in risk factors over time, such as changes in feeding policy, and seasonality in NEC incidence and mortality. Similar models have been applied to evaluate the health impact of smoke legislation^44^,^45^. Although the cohort was large enough for this type of analysis, a larger sample size could probably have narrowed the confidence intervals. As we could not account for the exact amount of breast milk administered in these infants, residual confounding in the infants receiving breast milk and formula (mixed group) may have occurred. It may be of interest to study the dose effects of breast milk in more detail in future trials. Another limitation of the study may be the relatively short duration of treatment, although it reflects current practice in our setting, as discussed earlier.

### Conclusions

In many centres over the world, probiotic use has become part of neonatal care to prevent NEC and mortality in preterm infants while questions on the optimal treatment strategy still remain. This study shows that the introduction of L. *acidophilus* and B. *bifidum* in our NICU was not independently associated with a decline in NEC or death over and above the underlying trend. A downward trend in the incidence of NEC was seen, but this could not be attributed to probiotics in the adjusted analysis. Our results suggest that the effects of probiotics may be modified by type of milk feeding in preterm infants, which should be considered in future studies.

## Additional Information

**How to cite this article**: Samuels, N. *et al.* Necrotising enterocolitis and mortality in preterm infants after introduction of probiotics: a quasi-experimental study. *Sci. Rep.* **6**, 31643; doi: 10.1038/srep31643 (2016).

## Supplementary Material

Supplementary Information

## Figures and Tables

**Table 1:** Baseline perinatal data and type of milk feeding.

Characteristics	Group 1 (n **=** 1288)	Group 2 (n **=** 673)	p-value
**Perinatal**			
Gender (male)	665 (52)	360 (54)	0.43
Gestational age (week)	29.4 (27.3–31.0)	29.6 (27.1–31.0)	0.96
Extremely preterm <28 weeks	421 (33)	213 (32)	0.64
Birth weight (g)	1163 (885–1425)	1175 (915–1453)	0.24
SGA	48 (4)	27 (4)	0.76
ELBW	468 (36)	235 (35)	0.53
Singleton pregnancy	968 (75)	526 (78)	0.14
Inborn delivery	1165 (91)	607 (90)	0.86
Ceasarean section	722 (56)	427 (63)	<0.01
Prenatal steroids^a^	1130 (88)	601 (89)	0.34
Mother prenatal antibiotics^b^	68 (5)	22 (5)	0.50
Infant direct postnatal antibiotics	1144 (89)	601 (89)	0.75
Apgar score at 5 min^c^	8 (7–9)	8 (7–9)	0.46
Cord arterial pH^d^	7.29 (7.23–7.33)	7.30 (7.23–7.35)	0.06
**Type of milk feeding**			
Only breast milk	63 (4.9)	151 (22)	<0.001
Mixed (breast milk and formula)	965 (75)	385 (57)	<0.001
Only formula	239 (19)	88 (13)	<0.01
ENS study milk	0	24 (4)	<0.001
No milk	21 (2)	25 (4)	<0.01

Shown are numbers (%) for categorical variables and medians (interquartile range) for continuous variables for patients born before (group 1) and after (group 2) introduction of routine probiotic treatment, and p-values.

Footnotes indicating number of missings: ^a^43 (35 and 8 per group respectively); ^b^204 (15 and 189, without clear explanation for difference); ^c^21 (9 and 12); ^d^not performed in 259 (142 and 117).

Abbreviations: CI, confidence interval; ELBW, extremely low birth weight; ENS: Early Nutrition Study milk (donor milk or formula); SGA, small for gestational age

Definitions: ELBW, defined as BW < 1000 g; SGA, defined as birth weight (BW) for GA < −2 SD.

**Table 2:** Clinical characteristics.

Characteristics	Group 1 (n **=** 1288)	Group 2 (n **=** 673)	p-value
**Probiotics**			
Probiotic treatment	0 (0)	626 (93)	NA
duration (days)	NA	11 (4–30)	NA
**NEC and sepsis**			
NEC ≥ stage 2	101 (8)	34 (5)	0.02
Surgical NEC	58 (5)	19 (3)	0.07
Age at diagnosis of NEC (days)	12 (7–19)	11 (6–23)	0.59
NEC after transfer	25 (2)	10 (2)	0.59
NEC related mortality	35 (3)	15 (2)	0.52
Any abdominal surgery	104 (8)	54 (8)	0.97
Suspected late onset sepsis	569 (44)	270 (40)	0.09
Blood culture-proven sepsis	264 (21)	126 (19)	0.35
Any IV antibiotic treatment	1218 (95)	624 (93)	0.10
**Respiratory support**			
Surfactant	610 (47)	345 (51)	0.10
Endotracheal mechanical ventilation	781 (61)	353 (53)	<0.001
duration of ventilation (days)	2 (0–8)	1 (0–6)	<0.01
CPAP or non-invasive ventilation	1084 (84)	579 (86)	0.27
**Nutrition and growth**			
Parenteral nutrition	1271 (99)	664 (99)	0.97
Reached full enteral feeding at NICU	646 (50)	257 (38)	<0.001
at day	12 (9–18)	13 (9–20)	0.14
Maximum weight loss (% of birth weight)	8 (3–11)	8 (4–11)	0.05
at day	3 (2–4)	3 (2–4)	0.20
Birth weight regained at NICU	737 (57)	378 (56)	0.66
at day	8 (6–11)	8 (5–10)	0.21
**Other**			
Length of NICU stay (days)	10 (4–26)	9 (4–25)	0.64
Umbilical artery catheter placed	384 (30)	137 (28)	0.46
Inotropic treatment	298 (23)	166 (25)	0.45
Red blood cell transfusion	586 (46)	206 (31)	<0.001
PDA treatment, medical surgical	305 (24)	143 (21)	0.22
	95 (7)	55 (8)	0.53
Intraventricular haemorrhage	196 (15)	82 (12)	0.07

Shown are numbers (%) for categorical variables and medians (interquartile range) for continuous variables for patients born before (group 1) and after (group 2) introduction of routine probiotic treatment, and p-values.

Data on NEC mortality were collected until day 120 (including time after NICU discharge), all other data shown relate to the NICU admission with a maximum of 120 days. NEC related mortality based on death certificates and medical records. Full enteral feeding was defined as an enteral milk intake of 150 ml*kg-1*day-1. No data were missing.

Abbreviations: CI, confidence interval; CPAP, continuous positive airway pressure; IV, intravenous; NA, not applicable; NEC, necrotising enterocolitis; NICU, neonatal intensive care unit; PDA, patent ductus arteriosus.

**Table 3:** Unadjusted associations between introduction of probiotics and each outcome.

Outcome measures	Group 1 (n **=** 1288)	Group 2 (n **=** 673)	OR (95%CI)	p-value
*Primary outcome*				
NEC or death	200 (15.5)	96 (14.3)	0.91 (0.69–1.18)	0.46
*Secondary outcomes*				
NEC	101 (7.8)	34 (5.1)	0.63 (0.42–0.93)	0.02
Mortality	148 (11.5)	78 (11.6)	1.01 (0.75–1.35)	0.95
Surgical NEC or death	180 (14.0)	84 (12.5)	0.88 (0.67–1.16)	0.36
NEC or sepsis or death	386 (30.0)	190 (28.2)	0.92 (0.75–1.13)	0.42

*Group 1:* patients born before introduction of probiotic treatment; *Group 2:* patients born after introduction of probiotic treatment. Shown are numbers (%), OR (95% confidence intervals), and p-values for unadjusted logistic regression.

Abbreviations: CI, confidence interval; NEC, necrotising enterocolitis; OR, odds ratio. Definitions: NEC, necrotising enterocolitis ≥ stage 2; NEC and all-cause mortality, recorded in the first 120 days of life; Sepsis, defined as blood culture proven late-onset sepsis during NICU admission.
