# Supplementary material for: Necrotising enterocolitis and mortality in preterm infants after introduction of probiotics: a quasi-experimental study
Source: Sci Rep. 2016 Aug 22;6:31643. doi: 10.1038/srep31643 (PMC4992873; doi:10.1038/srep31643)
Supplement: Supplementary Information [file srep31643-s1.pdf]

Supplementary Information

Necrotising enterocolitis and mortality in preterm infants after introduction of probiotics: a quasi-experimental study.

Noor Samuels, Rob van de Graaf, Jasper V. Been, Rogier C.J. de Jonge, Lidwien M. Hanff, René M.H. Wijnen, René F. Kornelisse, Irwin K. M. Reiss, Marijn J. Vermeulen MD PhD

Supplementary Table 1. Results of the primary analyses: Full multivariable interrupted time series logistic regression models for each outcome adjusted for interaction between probiotic introduction and type of milk feeding.

	Primary outcome		Secondary outcomes							
	**NEC or death**		**NEC**		**Mortality**		**Surgical NEC or death**		**NEC or sepsis or death**	
	OR (95%CI)	p	OR (95%CI)	p	OR (95%CI)	p	OR (95%CI)	p	OR (95%CI)	p
**Introduction of probiotics**										
Only breast milk	1.16 (0.33-4.01)	0.82	1.89 (0.28-12.7)	0.51	0.76 (0.21-2.71)	0.67	0.91 (0.26-3.21)	0.89	0.43 (0.21-0.93)	0.03
Mixed	1.04 (0.37-2.94)	0.95	0.74 (0.14-3.87)	0.72	1.30 (0.43-3.90)	0.64	0.99 (0.34-2.92)	0.99	2.21 (0.99-4.90)	0.05
Only formula	0.39 (0.10-1.53)	0.18	0.10 (0.08-1.36)	0.09	0.78 (0.19-3.17)	0.73	0.49 (0.12-1.99)	0.32	1.10 (0.40-3.03)	0.86
ENS study milk	Not estimable		Not estimable		Not estimable		Not estimable		Not estimable	
No milk	0.45 (0.07-2.85)	0.40	Not estimable		0.69 (0.11-4.33)	0.69	0.63 (0.10-3.98)	0.62	0.66 (0.12-3.57)	0.63
**Type of milk feeding (ref is only breast milk)**										
Mixed	0.44 (0.20-0.94)		1.63 (0.48-5.61)		0.28 (0.12-0.62)		0.39 (0.18-0.86)		0.47 (0.25-0.89)	
Only formula	1.08 (0.47-2.50)		2.90 (0.79-10.6)		0.77 (0.32-1.86)		1.01 (0.43-2.37)		0.67 (0.33-1.35)	
ENS study milk	0.76 (0.19-3.05)		Not estimable		0.75 (0.18-3.09)		0.87 (0.21-3.55)		0.61 (0.21-1.80)	
No milk	29.8 (6.54-136)		Not estimable		26.5 (5.92-119)		26.5 (5.88-119)		6.87 (1.72-27.4)	
**Male gender**	1.07 (0.80-1.43)		0.99 (0.68-1.44)		1.11 (0.80-1.54)		1.04 (0.76-1.40)		0.99 (0.80-1.24)	
**Prenatal steroids**	0.53 (0.34-0.83)		0.86 (0.47-1.57)		0.44 (0.27-0.71)		0.46 (0.29-0.73)		0.61 (0.42-0.87)	
**Gestational age (weeks)**	0.60 (0.56-0.64)		0.69 (0.63-0.75)		0.58 (0.54-0.63)		0.59 (0.55-0.63)		0.65 (0.61-0.68)	
**Birth weight Z-score**	0.87 (0.74-1.02)		-		0.81 (0.68-0.96)		0.85 (0.72-1.01)		0.81 (0.72-0.92)	
**Caesarean section**	1.44 (1.04-1.99)		1.46 (0.99-2.16)		-		1.50 (1.06-2.11)		1.51 (1.18-1.94)	
**Quartile 4 (each year)**	1.40 (1.00-1.95)		2.13 (1.40-3.25)		-		1.43 (1.01-2.03)		-	
**Quartile 1 in 2009**	-		-		-		-		0.56 (0.29-1.10)	
**Quartile 2 in 2010**	-		-		-		-		0.48 (0.24-0.96)	
**Quartile 3 in 2011**	0.42 (0.16-1.09)		-		-		-		-	
**Quartile 2 in 2012**	2.02 (0.93-4.42)		3.44 (1.38-8.55)		-		-		-	

Shown are adjusted associations between introduction of probiotics and each outcome according to feeding type; additional report on model presented in Figure 3.
Odds ratios indicate odds of developing each outcome in period after versus before introduction of probiotics, adjusted for the interaction between probiotic introduction and feeding type (reference is only breast milk), non-linear time trend (via B-splines; except for the outcome 'NEC or sepsis or death' where no time trend was present) and the variables shown. Cubic B-spline terms not shown.
Abbreviations: CI: confidence interval; ENS, early nutrition study; NEC, necrotising enterocolitis; OR, odds ratio; Ref: reference.
Definitions: NEC, necrotising enterocolitis stage ≥ stage 2; Surgical NEC, NEC requiring surgical treatment; Sepsis, blood culture proven lateonset sepsis during NICU admission.

Supplementary Table 2. Results of the post-hoc analyses: Full multivariable interrupted time series logistic regression models

	Host-hoc analyses			
	**NEC or death**		**Sepsis**	
	OR (95%CI)	p	OR (95%CI)	p
**Introduction of probiotics**				
Only breast milk	2.67 (0.70-10.2)	0.15	0.36 (0.12-1.09)	0.07
Mixed	1.47 (0.49-4.42)	0.49	1.40 (0.56-3.51)	0.47
Only formula	0.28 (0.07-1.19)	0.09	0.88 (0.27-2.83)	0.83
ENS study milk	Not estimable		Not estimable	
No milk	0.17 (0.02-1.16)	0.07	Not estimable	
**Type of milk feeding (ref is only breast milk)**				
Mixed	0.41 (0.18-0.91)		1.03 (0.50-2.12)	
Only formula	1.14 (0.48-2.75)		0.92 (0.42-2.03)	
ENS study milk	0.81 (0.19-3.43)		0.56 (0.17-1.87)	
No milk	40.9 (8.43-198)		Not estimable	
**Male gender**	1.00 (0.74-1.34)		0.93 (0.73-1.18)	
**Prenatal steroids**	0.53 (0.34-0.85)		0.95 (0.63-1.45)	
**Gestational age (weeks)**	0.54 (0.50-0.59)		0.72 (0.69-0.76)	
**Birth weight Z-score**	0.85 (0.72-1.01)		0.82 (0.72-0.94)	
**Caesarean section**	1.63 (1.16-2.28)		1.63 (1.23-2.14)	
**Treatment duration**	0.95 (0.94-0.97)			
**Quartile 4 (each year)**	1.40 (1.00-1.97)		-	
**Quartile 1 in 2009**	-		-	
**Quartile 2 in 2010**	-		-	
**Quartile 3 in 2011**	0.38 (0.15-1.03)		-	
**Quartile 2 in 2012**	2.02 (0.90-4.55)		-	

Shown are the adjusted associations between introduction of probiotics and each outcome according to feeding type in two post-hoc analyses: first, for the primary outcome including adjustment for probiotic treatment duration and second, the earlier used model for the outcome sepsis. Odds ratios indicate odds of developing each outcome in period after versus before introduction of probiotics, adjusted for the interaction between probiotic introduction and feeding type (reference is only breast milk), non-linear time trend (via B-splines; except for the outcome ‘NEC or sepsis or death’ where no time trend was present) and the variables shown. Cubic B-spline terms not shown.
Abbreviations: CI: confidence interval; ENS, early nutrition study; NEC, necrotising enterocolitis; OR, odds ratio; Ref: reference.
